# Towards Quantum-Chemical Modeling of the Activity of Anesthetic Compounds

**DOI:** 10.3390/ijms22179272

**Published:** 2021-08-27

**Authors:** Janusz Cukras, Joanna Sadlej

**Affiliations:** 1 Laboratory of Spectroscopy and Intermolecular Interactions, Department of Chemistry, University of Warsaw, Pasteura 1, 02-093 Warsaw, Poland; januszc@chem.uw.edu.pl; 2 Faculty of Mathematics and Natural Sciences, Cardinal Stefan Wyszynski University, Wójcickiego 1/3, 01-938 Warsaw, Poland

**Keywords:** anesthetics, xenon, intermolecular interactions, DFT, molecular dynamics, ONIOM, SAPT, lipid rafts, GABA_A_, NMDA, Gly, microtubules, TREK-1

## Abstract

The modeling of the activity of anesthetics is a real challenge because of their unique electronic and structural characteristics. Microscopic approaches relevant to the typical features of these systems have been developed based on the advancements in the theory of intermolecular interactions. By stressing the quantum chemical point of view, here, we review the advances in the field highlighting differences and similarities among the chemicals within this group. The binding of the anesthetics to their partners has been analyzed by Symmetry-Adapted Perturbation Theory to provide insight into the nature of the interaction and the modeling of the adducts/complexes allows us to rationalize their anesthetic properties. A new approach in the frame of microtubule concept and the importance of lipid rafts and channels in membranes is also discussed.

## 1. Introduction

The discovery of general anesthesia (GA) is a fundamental achievement for the development of contemporary biochemistry and biomedical science. No precise definition of GA has been established. Generally, as an answer to the question about anesthesia one can formulate three points: (i) amnesia, (ii) no response to a noxious stimulus and (iii) loss of consciousness [1]. In low concentration, the behavioral responses are well known: analgesia, amnesia, excitation, hypnosis and sedation, muscle relaxation, while reduced motor and autonomic response in higher concentration of anesthetics [1]. For anesthetics, several criteria have been put forward [2,3]; however, among them the most important is: anesthetics have to produce “a reversible effect at a functional site with clinically relevant concentrations”. 

Nowadays, it is still not known how anesthetic molecules cause reversible loss of consciousness. Neither do we know the mechanism of anesthesia nor how consciousness and memory arise in the brain. Therefore, the research in the field of anesthesia is one of the key paths towards understanding the phenomenon of consciousness [4,5,6,7,8,9].

Based on recent research it is now generally accepted that anesthetics act in multiple sites, and they modulate the synaptic transmission. However, a comprehensive experimental depiction of such action, e.g., in membrane proteins, is beyond the reach of modern experiment. The docking mechanisms and molecular dynamics (MD) simulations for anesthetics-protein receptors systems have become the methods of the first choice. MD description of trajectories contains useful information, e.g., about dominant conformations and average physicochemical properties in a given state. There have been a number of reports suggesting the importance of the intermolecular interactions which play an important role in the stability of proteins interacting with other molecules. This could be a prerequisite to understanding the anesthetic action.

As we have stressed above, the primary event of general anesthesia (GA) is the loss of consciousness. Here, we mainly emphasize the recent progress in the description of the molecular mechanisms of GA and the new concepts of the consciousness. Studying the interactions between general anesthetics and their macromolecular targets is crucial to the understanding of the biochemistry of these drugs. To identify the binding sites within targets and to determine novel binding molecules within a given biological system is the main aim of the investigations [4].

**Figure 1:**
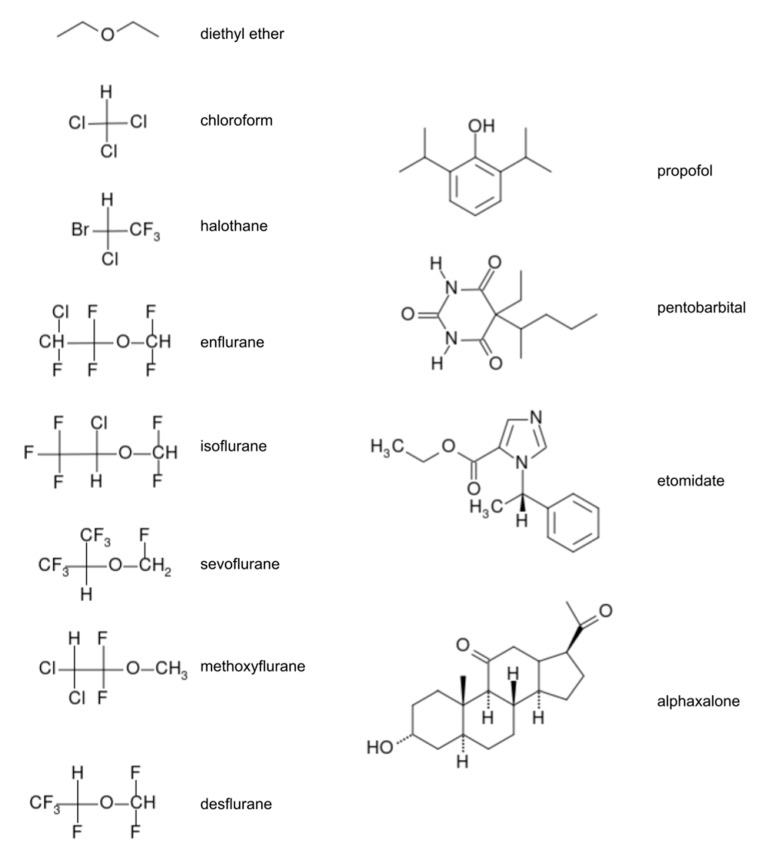
Structural formulas for most important general anesthetics.

Modern anesthetics are categorized into two main classes which are differentiated on the basis of the method of administration: inhaled (volatile) anesthetics and total intravenous anesthetics (TIVA) [3,4,6]. Inhaled anesthetics are: inorganic gases (nitrous oxide, xenon), multi-halogenated alkanes and ethers (e.g., halothane, sevoflurane (SEVO), isoflurane (ISO)). The halogenated alkanes are a challenge to computational description due to their unique characteristics as large halogenated compounds. Intravenous anesthetics usually contain more atoms (e.g., propofol and lidocaine) than volatile anesthetics and often have rings and multi-ring functionalities (e.g., ketamine and etomidate). The effect of GA is dose-dependent. Most popular GA are presented on Figure 1.

Within the central nervous systems (CNS) anesthetics can have many different targets. The so-called “unitary theory of general anesthesia” was popular in the early period [4] of investigations. According to this concept, general anesthetics act via a common molecular mechanism. At the same time, it was assumed that anesthetics depress neural activity of the CNS by roughly the same amount in all parts of the system. After a large number of experimental findings, however, the hypothesis of common mechanism was abandoned. In this review we summarize recent progress in the understanding of GA, and we pay particular attention to computational methods: the MD simulations and quantum-chemical models which describe intermolecular interactions.

The aim of this review is to summarize the last ten years of the computational studies on the anesthetic compounds and to compare, in terms of recently published outputs, the milestones in the development of the GA with the current achievements in elucidating the molecular mechanisms of anesthetic action. We adopt the point of view of molecular interactions theory and phenomenological framework. 

The review is organized as follows. In order to fully appreciate the issues surrounding the recently published new concept on anesthesia, we continue with a brief presentation of the milestones in the development of the anesthetics in Section 2: three relevant approaches known as “lipoid hypothesis”, “protein hypothesis” and “microtubule hypothesis”. We review the general anesthetics group of compounds with special attention paid to highlight the understanding of their exceptional characteristics. Afterwards, Section 3 describes shortly the computational methods used. Finally, Section 4 is dedicated to a description of the several results of anesthetics research in which computational techniques of classical molecular simulations and quantum-chemical methodology have played important roles, with special emphasis on the role of the intermolecular interactions (analyzed by changing the interacting partners) and the new concepts important to understanding the anesthesia.

## 2. Milestones in the Development of the Anesthetics: Lipid, Protein and Microtubule Hypothesis in General Anesthesia

### 2.1. Lipoid Hypothesis

The experimental parameter EC_50_ (concentration of half-minimal inhibition) is the concentration of a drug, with “stimulus-response” halfway between baseline and maximum after a specified time of exposure. It is equivalent to “the minimum alveolar concentration (MAC) parameter (expressed as vol%); the inhaled anesthetic atmospheric pressure required to prevent movement in response to a defined noxious stimulus in 50% of subjects”. Often, these values are of the order of the mM ranges. According to the experimental data one can notice a parallelism of binding and anesthetic effects.

In the frame of the first hypothesis of the mechanism of action of general anesthetics one can mention the Meyer–Overton rule [6,10,11]: “the logarithm of the efficacy of an anesthetic was related to the logarithm of its hydrophobicity” [6]. These findings supported the following interpretation: the lipid membrane could be the site of anesthetic action and implied that solubility in hydrophobic solvent is related to the anesthetic action. This work was a background for the ‘lipoid-like’ theory of anesthesia (the Meyer-Overton ‘lipoid’ hypothesis) and was named the ‘membrane-mediated mechanism’ [12,13].

The above idea considers anesthetic molecules as acting on lipids and membrane proteins of the CNS, before the changes within the CNS occur. Later, a few other unified theories have been proposed. Firstly, the pressure reversal was observed [4] and, secondly, stereospecificity of anesthetic action was observed. For instance, Harris et al. [14] found the influence of the stereospecific effects for the *S*(+)-isoflurane and *R*(−)-isoflurane in mice. Moreover, Franks and Lieb, refs. [15,16] showed that the Meyer-Overton correlation was also corresponding to anesthetics interacting with hydrophobic parts of proteins. This finding turned the attention to proteins as possibly involved in anesthetic action. Thus, two broad categories emerged in molecular research: one related to membranes and the other related to proteins/receptors and ion channels as candidates for the targets of anesthetics [4,13,17].

### 2.2. Protein Hypothesis

The second, hierarchical stage of the GA research [10,11] contains the description of the molecular level of the site and the mechanism of general anesthetic action, as well as the neural pathways and is usually named “protein hypothesis” [1,3,4]. It suggests that the anesthetic is acting specifically within hydrophobic pockets on certain proteins of membranes to produce the effect. Hence, the protein receptor hypothesis assumes protein as the target of general anesthetics and directs the attention towards interaction between anesthetics and allosteric sites of proteins.

It is generally assumed that amphipathic proteins and ion channels are under the influence of anesthetics and in this way the anesthetics modulate neural activity. They act as GABA (γ-aminobutyric acid) agonists and NMDA (N-methyl-D-aspartic acid) antagonists, or in neurons by changing membrane polarity. Ligand-gated ion-channels (sodium, potassium, calcium) have also been indicated as targets for many anesthetics [4]. Below, we summarize the most important information on the following four action sites: 

(i) The first one is the inhibitory γ-aminobutyric acid type-A (GABA_A_) receptor [4,18]. The GABA_A_ receptor belongs to a family of ligand-gated ion channels including the nicotinic acetylcholine receptor, the strychnine-sensitive glycine receptor and the 5-HT3 receptor. It belongs to the superfamily of the Cys-loop ligand-gated ion channels. Its isoforms comprise pentameric subunits with four transmembrane domains and a Cl^−^ channel in the center [18].

(ii) The glutamate NMDA receptors are the most probable sites of action [4,19]. The NMDA receptor is a hetero-tetramer consisting of two NR1 subunits and two NR2 subunits. To depolarize the cells, NMDA receptor allows the influx of Na^+^ and Ca^2+^ and the efflux of K^+^. NMDA receptors can become activated only in the presence of both glycine and glutamate and xenon’s anesthetic action in the CNS is probably related to xenon atoms competitively binding to the glycine binding site and thus inhibiting the receptor [19,20,21,22]. The NMDA receptor probably mediates the N_2_O action too [23,24]. However, other anesthetics, such as isoflurane, whose anesthetic target is GABA_A_ receptor, behave very differently. Three-dimensional atomic structures of the NMDA receptor are available [21].

(iii) The glycine receptor (GlyR) is an inhibitory receptor of the glycine neurotransmitter. It is an important target of inhaled anesthetics acting in the spinal cord [25]. It is a Cys-loop ligand-gated ion channel receptor. It has similar structural motifs to GABA_A_. Both receptors are inhibitory—regulating the membrane potential toward the Cl^−^ equilibrium potential. Similarly to GABA_A_, this receptor performs its inhibitory function by increasing the concentration of Cl^−^ ions inside the cell.

(iv) The two-pore potassium ion channels are crucial in restoring and maintaining resting membrane potential in excitable cells. Some studies have been conducted on K^+^ channels in tissues. The novel two-pore potassium channel subfamily member (K_2p_) have been suggested as important to GA. TREK-1 and TASK-1 channels (different types of K_2p_ channels) emerged as the main targets. TREK-1 is an anesthetic-sensitive K_2p_ channel that is activated by D2 (PLD2) (the enzyme phospholipase). Prior to the addition of anesthetic, PLD2 associates with the glycosphingolipid (GM-1) lipid rafts. Patel et al. [26] found that chloroform, diethyl ether, halothane and isoflurane activate TREK-1 and halothane and isoflurane activate TREK-1. 

In this framework, anesthesia is based on many different effects, and it consists in the interplay between the effects in proteins and the lipid bilayer, therefore one can name this group of facts as the “multiple-target hypothesis”. Various attempts to connect GA effect with proteins have been made. Since the structures of these receptors have not been established well, mutagenesis-based structure analysis is usually employed. There is no consensus on the protein mechanism of GA.

### 2.3. The Perturbation to Membrane and the Microtubules Concept, Targets of Interest

Recently, an extended hypothesis has been discussed: the perturbation to membranes. The involvement of the cellular membrane in GA is implicated from the conducted research but the mechanism is far from clear. Membrane and macromolecule system simulations are rapidly developing nowadays. Before the end of XX-century the membrane hypothesis was represented mainly by classical MD of phospholipid membrane. Nowadays one can distinguish three subgroups of large-scale simulations:

1. Membranes as targets: Classical “coarse-grain (CG) simulations” are used to study the interaction of GAs and model phospholipid membranes [27].
2. Microtubules as targets: The concept of microtubules inside neurons as anesthetic targets [28].
3. Lipid rafts as targets: it is assumed that anesthetics disrupt lipid rafts leading to production of phosphatic acid (PA) which is a signaling molecule [29].

Below, we briefly comment on these approaches, while the examples of the results will be presented in Section 4. 

The study of the interactions of anesthetic molecules by classical molecular dynamics simulations with model phospholipid membranes (fully hydrated 1-palmitoyl-2-oleoyl phosphatidylethanolamine (256 POPE molecules) started to be an active topic of research twenty years ago and is active until today [30]. Now, new approaches are used in the study as was mentioned above: (ii) the microtubules concept and (iii) lipid rafts and activation of phospholipase.

Microtubules consist of tubulin polymers which constitute part of the cytoskeleton. Studies have shown that some anesthetics can bind directly to tubulin, as in the case of halothane [28,31]. The authors found that “anesthetics can alter resonance in π-electron cloud oscillations among highly polarizable non-polar amino acids in tubulin”. Recently, Tuszynski group [28,31] have shown a very strong correlation between the potency of an anesthetic and the shifts it induces in the collective “electronic cloud oscillations”.

In point (3) we grouped the Cys-loop family of pentameric ligand-gated ion channels (pLGIC), which are affected by anesthetics. The Cys-loop receptors are well described in terms of GA. It has been assumed that the channel, which is located in a raft, is activated by change in its environment which is brought upon by an anesthetic molecule or molecules disrupting the lipid surroundings of the channel. Recently, it was shown [32] that mechanical force disrupts phospholipase D2 (PLD2) localization, and that the disruption activates the production of signaling lipid phosphatidic acid (PA).

## 3. Methods of Calculations

Two main points related to the theoretical study of the anesthesia remain a challenge: (i) the vast complexity of the mechanism; it seems to be multi-faceted and that many molecular pathways might be involved; therefore, an efficient method to model huge and complex molecular systems consisting of many large and small molecules is required; (ii) the intermolecular interaction description at the quantum level from the perspective of both electron densities interaction and spin interaction.

Three methods of calculations seem to be particularly important in the context of anesthesia, each with its unique advantages and setbacks: molecular dynamics (MD) which is based on classical mechanics, and two quantum-mechanical approaches: Density Functional Theory (DFT) [33] and Symmetry-Adapted Perturbation Theory (SAPT) [34]. 

Historically, because of the demands related to computer power, quantum-chemistry methods have been limited to small systems but is has to be noted that recent advances in computer technology as well as theory and code development allow for increasingly large systems to be treated with quantum mechanical methodology, e.g., with linear-scaling methods, as implemented in LSDalton [35,36]. Recently, many hybrid methods have been developed, e.g., Polarizable Embedding Quantum Mechanics/Molecular Mechanics (PE QM/MM) [37] which describes part of the system at the quantum level and the other part with classical physics.

As far as intermolecular interaction energies are considered, there is a large quantum-chemical database of model complexes available for reference. Řezáč et al. calculated interaction energies using accurate methods in complete basis-set limit for 66 different complexes which provide an important reference point for anyone studying intermolecular interactions [38].

### 3.1. MD: Classical-Physics Approach to Molecular Systems

Molecular Dynamics (MD) is based on the Molecular Mechanics (MM) approach to study molecular systems using classical mechanics equations based on Newton and Hooke’s laws. This choice made it possible to run simulations in the domain of time for systems which are completely out of the scope of any modern quantum-chemical method. Due to the lack of accurate quantum chemistry description force fields need to be used which have to be developed prior to simulation.

MD became widely used in all the areas where structural and dynamical insights are needed to understand biological molecular systems. Application of the method has been very successful, from enzyme dynamics to docking and ligand interaction. They elucidated many mechanisms of processes involving proteins and nucleic acids. Statistical analysis of the simulation can provide thermodynamics of the system [39]. With the use of force fields, however, a host of parameters and approximations emerge which makes it difficult to control the simulation and to interpret the results. For instance, hydrogen bonds which fundamentally have quantum and chemical nature are described by means of potentials around point charges. It seems that proper modeling of anesthetics interacting with biological macromolecules calls for the use of quantum mechanical description, especially in the light of recent findings [28,40,41,42]. 

### 3.2. Quantum-Chemical Methods

#### 3.2.1. DFT: A Quantum-Chemical Method for Large Systems

The well-known Density Functional Theory (DFT) is different from other quantum-chemical methods in that it describes the molecular system in terms of functionals of electron density instead of orbital wavefunctions which results in huge computer power reduction—the times scales of typical DFT calculations are comparable to Hartree-Fock, which is the most fundamental quantum-chemical method based on the wave function formalism [43]. Thus, it proved useful in the studies of large molecules while allowing for quantum description of the system despite the problem of the approximated exchange-correlation functional (e.g., B3LYP, CAMB3LYP, LDA etc.).

The system sizes which are of interest in the study of anesthesia are accessible by the DFT method but, while the frontier is constantly moving further, its practical use today is quite limited to structure and properties of frozen molecules. Still, one needs to note that fast advancements are made and the DFT method is very promising for future use in dynamics-based computational biochemistry as is evidenced in the recent calculations of protein-ligand binding free energies by means of DFT/QM-PBSA method, performed for over 2000 atoms, with sampling of 10 to 100 snapshots [44].

Quantum-chemical methods provide a much better description of the interactions between species which are not covalently bonded than those based on classical physics. Recent findings on the importance of quantum-level description of the anesthetic action even further underscores the necessity to use such methods [44]. While one can obtain accurate interaction energy estimates by the use of DFT, there are even more sophisticated approaches to intermolecular interaction energy offered in the field of computational chemistry.

#### 3.2.2. SAPT: A Method Which Could Provide New Understanding of Anesthesia

The common way of calculating the intermolecular interaction energy of a complex is to calculate its total energy and subtract the sum of the monomers’ energy:

(1) $$\Delta E=E_{\mathrm{tot}}-\overset{N}{\underset{i=1}{\sum }}E_{i}$$

Treating the complex as a supermolecule gives the method the name “supermolecular”. This solution poses some methodological problems, e.g., since the monomers by default have different basis sets than the complex, a special procedure is needed to avoid the so-called Basis Set Superposition Error (BSSE). The total energies of molecular systems are orders of magnitude larger than the intermolecular interaction energies, so the total energies need to be calculated with appropriate convergence criteria. This method provides no information about the interaction beside its magnitude.

Another quantum-chemical approach to describe the interaction in a complex is a perturbative one. Here, the interaction is treated as a small perturbation to the total energy of the complex. An early approach was Rayleigh-Schrödinger perturbation theory, but it lacked the description of the repulsion at close distances. This problem was solved by enforcing the Pauli principle with antisymmetrization of the dimer wave function yielding SAPT [34].

SAPT allows for calculation of the intermolecular interaction energy term-by-term and, as for now, is limited to two-body and three-body interactions. Since the perturbational approach provides mathematical expressions which yield physical interpretation, we can infer about the nature of the interaction between constituents of the complex. SAPT is BSSE-free, and the number of computed terms can be chosen depending on the needs. The common SAPT analysis levels are called SAPT0, SAPT2 and SAPT which include electron correlation to different extents. Another advantage of the perturbative framework is the ability to calculate different terms with different basis sets, e.g., one could calculate the induction energy and the dispersion energy each with its own basis set required for proper description [45]. 

Generally, the SAPT expansion for the interaction energy can be expressed as follows:

(2) $$E=\overset{\infty }{\underset{n=1}{\sum }}\overset{\infty }{\underset{i=1}{\sum }}\left(E_{\mathrm{RS}}^{\left(ni\right)}+E_{\mathrm{exch}}^{\left(ni\right)}\right)$$

where $E_{\mathrm{RS}}^{\left(ni\right)}$ denotes the Rayleigh-Schrödinger terms and $E_{\mathrm{exch}}^{\left(ni\right)}$ denotes the exchange energy terms that arise due to antisymmetrization of the wave function. *n* is the order of the term with respect to perturbation operator and *i* is the order with respect to intramonomer correlation operator. The many terms that arise from the SAPT expansion can be roughly collected into following contributions (for notation, see [46] and references therein):

(3) $$E_{\mathrm{elst}}=E_{\mathrm{elst}}^{\left(10\right)}+E_{\mathrm{elst},\mathrm{resp}}^{\left(12\right)}+E_{\mathrm{elst},\mathrm{resp}}^{\left(13\right)}$$

(4) $$E_{\mathrm{ind}}=E_{\;\mathrm{ind},\mathrm{resp}}^{\left(20\right)}+{\;}^{t}E_{\;\mathrm{ind}}^{\left(22\right)}$$

(5) $$E_{\mathrm{disp}}=E_{\mathrm{disp}}^{\left(20\right)}+E_{\mathrm{disp}}^{\left(21\right)}+E_{\mathrm{disp}}^{\left(22\right)}\;$$

(6) $$E_{\mathrm{exch}}=E_{\mathrm{exch}}^{\left(10\right)}+\epsilon_{\mathrm{exch}}^{\left(1\right)}\left(\mathrm{CCSD}\right)+E_{\;\mathrm{exch}-\mathrm{ind},\mathrm{resp}}^{\left(20\right)}+{\;}^{t}E_{\mathrm{exch}-\mathrm{ind}}^{\left(22\right)}+E_{\mathrm{exch}-\mathrm{disp}}^{\left(20\right)}$$

Four main contributions can be defined and interpreted, namely (1) electrostatic energy, *E*_elst_, (2) exchange energy, *E*_exch_, (3) induction energy, *E*_ind_, and (4) dispersion energy, *E*_disp_. The electrostatic energy is understood as the interaction between permanent multipoles of both constituents. The exchange energy arises from the Pauli principle and is always repulsive. The induction energy is the interaction between permanent multipoles of one of the monomers with the induced multipoles of the other monomer and vice versa. It corresponds to charge shift taking place due to complexation. The dispersion energy comes from the interaction of induced multipoles of both constituents. Thus, the composition of the interaction energy reflects the properties of the interacting species and gives a detailed description of the complex in the frame of the language of physics. In the study of anesthesia, investigation of the nature of the interaction of anesthetics is largely missing but it could provide interesting, if not crucial, insights. 

The SAPT method is implemented in many code suites, the most prominent being SAPT2020 [46], PSI4 [47] and MOLPRO [48]. All of them implement two-body SAPT. Additionally, SAPT for the calculations of the three-body corrections to nonadditivity is available in a separate code (sapt3b) by Szalewicz et al. [46] and it computes only the three-body effect in the interaction of three molecules, i.e., the two-body interactions still need to be calculated using the two-body SAPT. PSI4 deserves special attention since its versatile implementation allows for interacting with it as a Python module, e.g., from within a Jupyter notebook, and it implements intra-molecular SAPT. Also, PSI4 implements the so-called F-SAPT, which allows for analysis of the intermolecular interaction energies divided spatially into different parts of the interacting molecules [49,50].

The SAPT method is also implemented at the DFT level, the so-called SAPT(DFT) [51,52], and even further extended with density fitting [53] which paved the way for applications in larger systems. Still, the methodology of SAPT is quite expensive and its use for systems as large as proteins is not feasible yet. One needs to approximate protein with its fragments which interact with agonists/antagonists or other species. 

## 4. Selected Results of the Anesthetic Calculations 

The used anesthetics may be arranged in three groups A, B, C according to clinical features and molecular targets. The A group contains: weak hypnotics and potent analgesics such as Xe and N_2_O, to which the central mechanism is the inhibition of the N-methyl-D-aspartate (NMDA). The second group, B, formed by strong hypnotics and amnestics such as halogenated ethers and alkanes, interacting with GABA_A_, glycine receptors and 2-pore K^+^ channels. The third group, C, contains strong hypnotics and weak immobilizers such as propofol and etomidate interacting with GABA_A_ receptors and the subtypes. The results presented in this chapter are examples of the MD as well as the quantum-chemical methodology.

### 4.1. Xe, N_2_O 

The properties of xenon make it an ideal volatile anesthetics. Xenon is not a greenhouse gas and has no known detrimental influence on the environment contrary to the other inhaled anesthetics, as chlorofluorocarbons and nitrous oxide. In Earth’s atmosphere it occurs as a trace element [41,54,55,56,57,58,59]. Its blood/gas partition coefficient is as low as 0.15, it has a MAC of 63%, which results in rapid induction of the anesthetic effect and equally rapid recovery from it. Xenon provides excellent cardiovascular stability. It induces anesthesia in different species, including *Drosophila*, mice, and humans [40,41,60]. Very recently, argon was also found to form stable adducts in several van der Waals systems [55,59]. The electronic closed-shell nature of the noble gas plays the main role in the description of the energy components involved in the intermolecular forces of such complexes, underscoring the noncovalent interactions.

**Figure 2:**
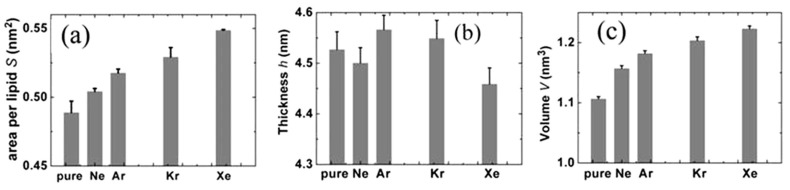
Noble gas molecules on bilayer structural properties: (**a**) area per liquid, (**b**) thickness and (**c**) volume per liquid; error bars mean the root mean square deviations (reproduced from ref. [59], Creative Commons 4.0 licence).

MD simulations were performed on 1-palmitoyl-2-oleoyl phosphatidylethanolamine (256 POPE molecules) bilayers in aqueous solution (40 water molecules per lipid) with or without noble gas atoms [30]. The electronic closed-shell nature plays the main role in the description of the components involved in the intermolecular forces. In these extensive MD simulations of POPE membranes the atoms were approximated with uncharged Lennard-Jones (LJ) spheres. Universal force field (UFF) was employed for their van der Waals (vdW) parameters. A sequence of Ne, Ar, Kr and Xe was established in terms of the effect on POPE from the weakest to the strongest, which is in good agreement with the anesthetic efficacy of these gases. More xenon atoms were distributed among the lipid molecules than other noble gases which influenced the arrangement of the lipids in the membrane. This seems to correspond to xenon’s strong anesthetic potency. Also, this result supports the membrane mediated mechanism hypothesis. Similar results were found by Koziakova et al. [58]—xenon and argon are equally protective against hypoxia-ischaemia in vitro; however, Xe action is mediated by the glycine site of NMDA receptor, whereas argon does not inhibit NMDA, but probably acts via a different pathway [55,59,61]. Figure 2 shows three parametrs for four gases: area per lipid *S* (on the left), thickness *h* per lipid (in the middle) and volume V = S*h/2 per lipid (on the rigth). Two of the three parameters *S* and *V* change in the order of Ne, Ar, Kr and Xe, corresponding to narcotic potencies, while the *h* parametr does not hold this trend. Xenon is characterized by smaller parametr *h* compared with pure membrane [55,59,61].

In terms of the distribution of atoms in the membrane, xenon is unlike the other noble gases and resembles other anesthetics, such as chloroform and enflurane, which again seems to contribute to its strong anesthetic efficacy. Similar MD calculations were performed for Xe and lipid bilayer by manipulating the pressure to answer the question on the mechanism of the pressure reversal in GA [56]. The results of the simulation suggest that the pressure reversal is caused by hindered mobility of the xenon atoms within the membrane. However, if we assume the Meyer-Overton rule and the Franks-Lieb protein hypothesis to be true, there should be one common mechanism for all GAs, including xenon.

Xenon is a NMDAreceptor antagonist. The investigations of xenon with the ligand-binding domain of a NMDA receptor were performed by Liu et al. [57] and Andrijchenko et al. [61]. The authors of [57] performed several MD simulations on the open and closed cleft ligand-binding domains (LBDs) with and without the Xe atom. They found that Xe could weaken the agonist binding, but it gives more profound effects on the LBD in different cleft configurations. To summarize, MD simulations showed decreased glutamate binding which could lead to low agonist efficacy and competitive inhibition mechanism [57]. 

**Figure 3:**
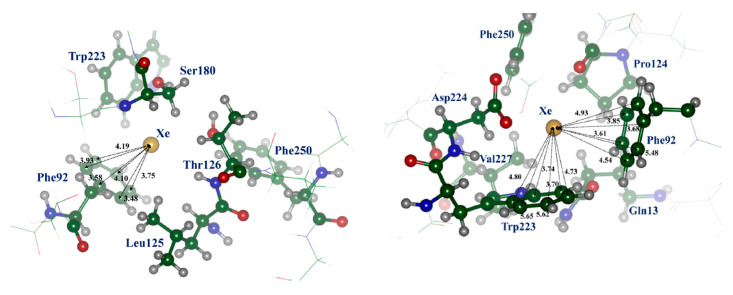
Structure I (on the **left**) and II (on the **right**) of the trapping site Xe···NMDA; the xenon atom forms a typical π-type complex with the aromatic ring of Phe92 or Phe92 and Trp223; C atom—green, N—blue, O—red. (Adapted with permission from [61]. Copyright 2015 American Chemical Society).

More advanced calculations were performed by QM/MM (QM/DFT/D2/MM, D2 meaning dispersion correction) and molecular dynamics with the Lennard-Jones interaction potential (MD) methods by Andrijchenko et al. [61]. It was explored whether xenon can play a role as an antagonist of the NMDA receptor inhibiting the receptor at its glycine binding site. The typical model of π-type complex of xenon atom with aromatic moiety was taken into account. Figure 3 presents such interaction of Xe atom with Phe92 (structure I) and Phe92 and Trp223 (structure II). The simulation showed that xenon atoms can compete with glycine molecules for binding on the protein receptor. 

**Figure 4:**
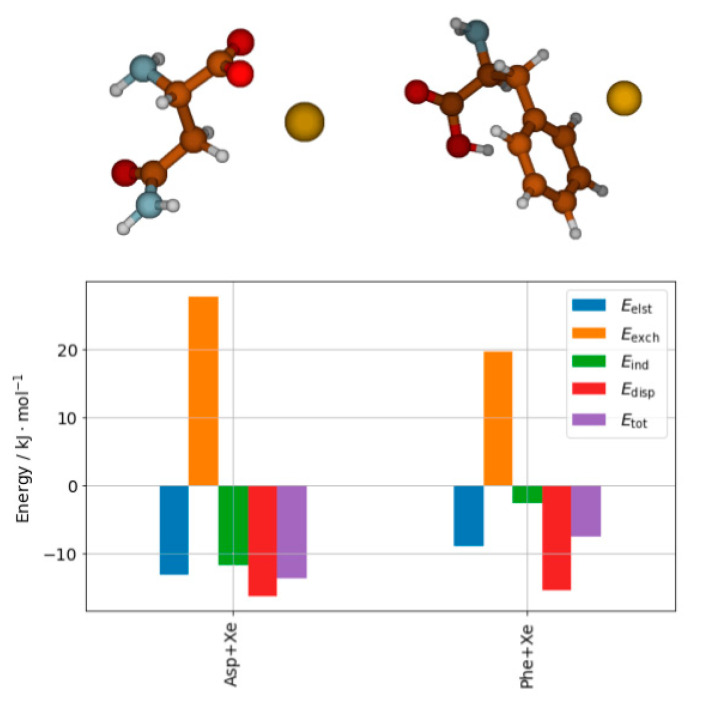
Structures of the complex of asparagine with Xe atom (Asp+Xe) and the complex of phenylalanine with Xe atom (Phe+Xe) and the SAPT decomposition of their interaction energy [62].

A detailed description of the interaction can be obtained from SAPT analysis [62]. Recent results performed at the SAPT0/aug-cc-pVDZ level with relativistic effects on xenon taken into account by means of effective core potentials (ECP) show a completely different picture of the nature of the interaction depending on the site of the interaction. The results are shown in Figure 4. In both cases the electrostatic energy is compensated by the exchange interaction, so the induction and dispersion contributions are what keeps the complexes stable. Moreover, one can clearly see that the ratio of dispersion to induction energy differentiates both complexes. The role of dispersion relative to the total SAPT interaction is much larger in the case of phenylalanine, while the induction has the dominant role in the case of asparagine complex. 

**Figure 5:**
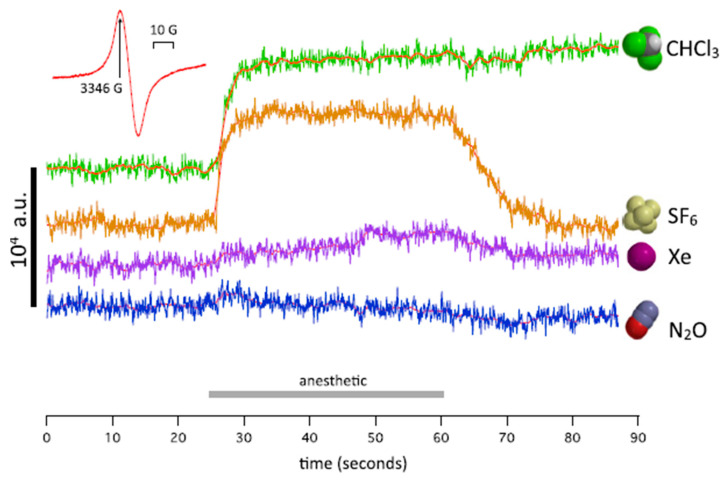
Four traces of measurements of spin at a fixed value of magnetic field (*Inst*; 3346 G). The order of exposure was Xe, SF_6_, N_2_O, CHCl_3_. No signal is present in N_2_O and a small signal is in Xe (Reproduced from ref. [41]).

Recently, it was shown that xenon produces anesthetic effects due to unpaired-electron transfer as well as nuclear-spin dependence. These investigations seem to be pioneering. A series of papers [41,63,64,65] was published, which present a new approach to the physical information and thus a new physical mechanism of anesthesia itself. Firstly, Turin et al. [41] described the electron-paramagnetic-resonance (EPR) measurements showing an induced spin change for the *Drosophila* flies treated with anesthetic. The results were obtained for Xe, N_2_O, CH_3_Cl [41], see Figure 5. The authors confirmed that anesthetic-resistant mutant strains of “*Drosophila* exhibit a different pattern of spin responses to anesthetic”.

The EPR experiment was supplemented by the DFT calculations. The calculations supported the conclusion that the observed changes are caused by perturbation of the electronic structures of the proteins by anesthetic molecules. Moreover, Turin et al. [41,66] found that no change of spin was detected in the *Drosophila* if no oxygen was added to xenon in the experiment. 

Secondly, Li et al. [40] have been investigating the influence of nuclear spin on GA which may help to elucidate the quantum mechanisms involved in anesthetic action and consciousness. Using the DFT/B3LYP method, the polarizabilities of four isotopes were calculated with the aim to compare effects of xenon isotopes ^129^Xe (I = 1/2), ^131^Xe (I = 3/2), as well as ^132^Xe and ^134^Xe having the nuclear spin I = 0 on their anesthetic potencies. The experimental anesthetic potency in animals measured by “loss of righting reflex ED_50_ and tail clipping” was compared with the calculated results. The main result of these comparisons is the conclusion: the anesthetic potency of xenon isotopes with nuclear spin not equal zero is less than the anesthetic potency of two xenon isotopes that have nuclear spin I = 0. This difference in anesthetic potency cannot be explained by the differences in outer electron shells or the variations in atomic masses of the atoms or the polarizability, which is the same (3.60 Å^3^) for the isotopes. The authors suggest that the “quantum property” of nuclear spin in the xenon atom is related to consciousness processes at the xenon site of action.

The next recently published paper by Smith et al. [63] discusses hypotheses on xenon-induced anesthesia in the frame of the Radical-Pair Mechanism of electrons (RPM) by taking into account the hyperfine interactions. The RPM is a phenomenon described in laboratory studies of radical reactions [64]. Spin multiplicity is conserved when two radicals are produced simultaneously, and the spins of the unpaired electron are correlated with each other for a time, in a singlet or triplet configuration. If each unpaired spin experiences a different local magnetic field, then the singlet and triplet spin states will interconvert. The rate of interconversion is influenced by the magnetic field. In the case of xenon-induced anesthesia the RPM Hamiltonian depends not only upon the number of xenon atoms in the binding site of the receptor, but also on the hyperfine interactions and thus on the hyperfine coupling constant. 

To determine the hyperfine interactions between the radicals and xenon atoms and to reproduce the experimental isotope-dependent anesthetic effects Smith et al. [63] studied the xenon-NMDA receptor system based on the xenon atoms surrounded by phenylalanine and tryptophan residues located in the glycine-binding site of the NMDA receptor by means of MM and DFT. It has been suggested that the information on the magnetic field direction might be transferred into chemical signals. A model of the magnetoreception (observed in the case of birds) involves the cryptochrome protein [64,65,67]. 

The second compound, nitrous oxide, N_2_O, volatile anesthetic (VAs), commonly known as “laughing gas”/”happy gas”/“ageing gentleman” of anesthesia [68,69,70,71,72], has many advantages and disadvantages. It has a very weak anesthetic potency, having a MAC of 104. Therefore, it is used in high doses (60% vs. 2% for sevoflurane) and as an aid to other anesthetics such as propofol or sevoflurane. It is the most commonly used inhalational anesthetic in dental procedures. Several studies show the influence of N_2_O on EEG signals in patients and volunteers [73]. It is also often combined with other anesthetics as a carrier gas. It releases proenkephalin in the CNS. Nitrous oxide has detrimental effect on the environment and its concentrations are regulated.

N_2_O acts as NMDA receptor antagonist [69,70,71,74,75]. In this respect it differs from the other volatile anesthetics which influence the GABA_A_ receptors and inhibit potassium channels in neurons (TREK-1), a two-pore-domain channels expressed throughout the CNS, among other targets [74,76,77]. Nitrous oxide together with other GABA_A_ modulators act in synergy evoking amnesia and hypnosis. Moreover, nitrous oxide acts on the opioid system which could contribute to analgesia and psychotropic effects [70,71,73,78,79]. It is also a weak antagonist of serotonine-type [77,80] receptors, and is a partial inhibitor of some nicotinic acetylcholine receptors [77]. Some investigations show that this compound exerts stronger effects on the κ-type opiate receptor than μ-type receptor [76,77].

Quantum-chemical investigation of different complexes which contain nitrous oxide interacting with other molecules are not frequent; among them are systems: N_2_O·H_2_O [81], N_2_O·N_2_O [82] and N_2_O·SO_2_, N_2_O·N_2_O, (N_2_O)_2_·SO_2_ [83]. Their conformers and spectroscopic constants were found and compared with the experimental observations. The potential energy surface quantum-chemical calculations by Coupled-Cluster Singles-Doubles with perturbative Triples method (CCSD(T)) with basis set aug-cc-pVTZ predicted four conformers for N_2_O dimer, while experimentally only two of them are known to date [82]. The infrared spectra (IR) of the polar dimer which correspond to the out-of-plane antisymmetric stretching of both isomers were observed. Heterodimer N_2_O·H_2_O [81] was studied at a lower level of theory and small shifts in the frequencies and the changes in intensity of H_2_O and N_2_O bands upon dimerization (N_2_O·H_2_O → (N_2_O)_2_) were found which suggests weak interactions in the complex. However, the investigations of the interaction of N_2_O with peptides or at least amino acids are still an unexplored area. 

### 4.2. Inhaled Halogenated Anesthetics

Anesthetics are arranged in two main categories differentiated by the delivery method: inhaled (volatile) anesthetics and intravenous anesthetics. Most inhaled anesthetics have many halogenated atoms (e.g., halothane and desflurane), while the intravenous subgroup has larger molecules (e.g., propofol and lidocaine) usually with ring and multi-ring moieties (e.g., ketamine and etomidate) [30].

Two groups of methods were used to study the inhaled, halogenated anesthetics: quantum-chemical methods in the frame of the DFT or SAPT calculations and MD simulations. The first group of methods provides insight into the structure of possible binding sites of anesthetics, while the modulation of channel function requires a dynamic description of the anesthetic/ligand-gated ion channels (pLGIC) complex by MD simulation.

#### 4.2.1. Molecular Dynamics Simulations 

Many studies have been motivated by the acceptance of the nonspecific membrane disruption mechanism proposed by Meyer-Overton based on the discussed correlation between the solubility of anesthetic molecules in oil and their potency. There are many published articles examining the role of the lipid membrane by means of MD simulations [27,29,30,84,85]. One can divide them into two main groups:

(a) Some of them have proposed that anesthetics alter specific membrane properties.
(b) Supported by recent experimental spectroscopic measurements and crystallography, as well as MD simulations, one can distinguish a second group of results which demonstrate that the binding of an anesthetic specifically to ion channels is important to understanding anesthesia.

In recent years a hypothesis about the direct binding of anesthetics to pentameric ligand-gated ion channels (pLGICs) or Cys-loop nicotinic acetylcholine and γ-aminobutyric acid class A receptors, as well as the voltage-gated sodium and potassium channels, and the tandem pore potassium channels was proposed. The pLGICs are crucial to living organisms because they are responsible for converting chemical information into electrical signals within the nervous system.

Among dozens of articles on MD simulations of the inhaled anesthetics we choose here a couple of the most interesting examples from the point of view of the results as well as the used methods, articles which belong to the first and the second group of results. Since, here, we focus on recent research, we will only mention one study belonging to the first group, namely the study by Arcario et al. [30]. In this study the authors present a novel approach for the inhaled anesthetics: desflurane, isoflurane, sevoflurane, and propofol (Figure 1), which is compatible with the CHARMM (Chemistry at HARvard Macromolecular Mechanics) [86] force field for biomolecules. The 1-palmitoyl-2-oleoyl-*sn*-glycero-3-phosphatidylcholine (POPC) bilayer was used for modeling the interaction with the membrane in frame of the membrane change concept. These studies support previous works and show that the energy minimum is ca. 20 kJ/mol at the interface between the tail region of the lipids and their glycerol moiety. The determined membrane structural parameters (atomic distribution, dipole potential) after flooding the membrane with anesthetics show that the anesthetics do not alter the lipid bilayer. This observation leads to the conclusion that “an indirect membrane-mediated mechanism of channel modulation is unlikely” [30]. This study suggests that chosen anesthetics do not affect membrane structure.

From the second group, one can discuss an article on sevoflurane, an anesthetic which is one of the most popular, although recently it was found that sevoflurane may also affect cognitive function [87]. Stock et al. [88] demonstrated that this anesthetic binds to the voltage-gated potassium channel K_v_1.2. From experimental work it is known that sevoflurane “potentiates the channel in a dose-dependent manner”. Therefore, the following question was formulated: does sevoflurane shift channel equilibrium by binding to the open and closed protein structures depending on the conformation? With the help of the advanced and innovative MD simulations (extensive docking and free-energy perturbation of all binding sites) it was found that sevoflurane binds open and closed structures at multiple sites when saturation and concentration is taken into account. This case illustrates the very interesting example of multiple binding of anesthetic to ion channels K_v_1.2 and, moreover, is in agreement with the experimental measurements [88].

Second example from the ion channel mechanism is the study on fentanyl. Fentanyl belongs to the opioids and is much more potent than morphine [89]. It induces anesthesia in combination with the general anesthetic propofol [90]. Propofol, an intravenous anesthetic, was recently described in a few papers [90,91,92]. The mechanism of the potentiation of propofol is still unclear. The cited case is the first attempt to describe interaction of two different anesthetics in GA [90]. To study these drugs interacting with the *Gloeobacter violaceus* ion channel (GLIC), classical MD using “flooding style” and Gaussian accelerated Molecular Dynamics (GaMD) simulations were used. The interaction between fentanyl and propofol started with the “pore-blocking” mechanism by propofol and the formation of small clusters, which diffuse into the membrane region. Here, a number of propofol molecules leave the fentanyl, while other propofol molecules migrate to the extracellular part of GLIC, to the region of orthosteric binding site [90]. The results demonstrated that fentanyl stabilizes the interaction of propofol with the ion channel. Such conclusions could not have been reached before with the use of conventional MD simulation methodologies.

TREK-1 is one type of the anesthetic-sensitive two-pore-domain potassium (K_2p_) mechano-gated channels occurring in mammalian neurons. It is an established target of inhaled anesthesia [93]. TREK-1 channels are very important in physiological and pharmacological processes. Before the action of the anesthetic, the phospholipase enzyme (PLD2) binds to GM-1 lipid rafts. Then it activates TREK-1 by binding to a C-terminus and produces high local concentrations of the phosphatidic acid (PA). Xenon, diethyl ether, halothane, and chloroform all can substantially activate TREK-1 at practical concentrations.

#### 4.2.2. Quantum-Chemical Investigations: Molecular Complexes of Inhaled Anesthetic with the Electron-Donor Compounds 

A deep understanding of the molecular interactions among the molecules taking part in the anesthesia is critical in the interpretation and in advancing chemistry of the anesthetic compounds. As anesthetics act in hydrophobic lipid-like and water environments, the intermolecular interactions include electrostatic forces due to permanent and induced dipole moments as well as the quantum mechanical interactions like dispersion forces (van der Waals interactions).

In order to highlight the intermolecular interactions, we decided to mention the papers which study the molecular complexes containing the halogen and hydrogen bond by quantum-chemical methods. First, we discuss the papers published in the beginning of the XXI century, which were limited by the early computational software and hardware available at the time, as well as the lack of the availability of anesthetic-protein X-ray structures. Secondly, we present the problems studied by the contemporary SAPT approach and Own N-layer Integrated Molecular Orbital Molecular Mechanics (ONIOM) [94,95] methodology.

**Figure 6:**
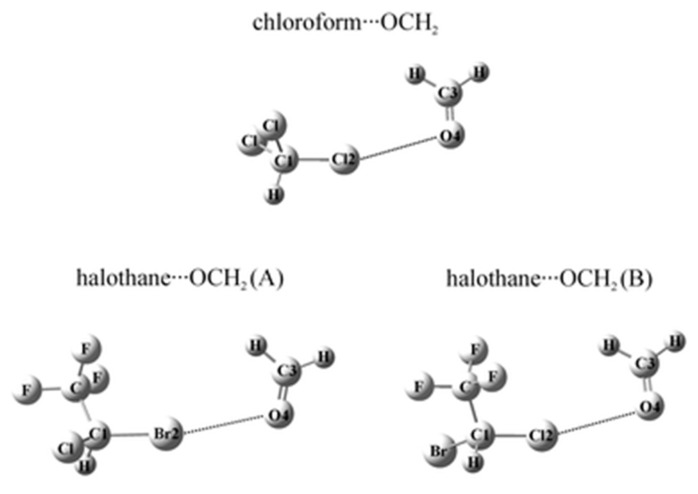
Optimized structures of chloroform···formaldehyde and two halothane···formaldehyde (**A**,**B**) complexes calculated at the MP2/6-311++G(d,p) level (Republished with permission of Royal Society of Chemistry, from [96], Copyright 2011; permission conveyed through Copyright Clearance Center, Inc.).

The halogen bonded complexes of several anesthetics (chloroform, halothane, enflurane and isoflurane) with formaldehyde were studied by ab initio second-order Møller-Plesset Perturbation Theory (MP2) and Coupled-Cluster Singles-Doubles with Perturbative Triples (CCSD(T)) methods [96,97,98] and the Natural Bond Orbital (NBO) analysis was applied at the MP2/6-311++G(d,p) level of theory. In the structures of these halogen bonded systems one can find the intermolecular halogen bond (see Figure 6). It is described as a noncovalent interaction X···O between a covalently bound halogen of one monomer and a negative site of the other. Halogen bonds resemble hydrogen bonds [99]. The obtained structures calculated on the MP2 or CCSD(T) level are the result of the balance between the energy components. It was found that the C–Br···O bonded halothane···OCH_2_ complex is the most stable. The electrostatic and dispersion terms of SAPT constitute about 95% of the total interaction energy of these complexes.

A region of negative electrostatic potential around the part of the lone pairs of the halogen atom bonded to the carbon atom forms the so-called sigma-hole, positive electrostatic potential (ESP). According to the results, the C-X bond lengths (X= Cl, Br) are contracted due to the Pauli exchange overlap repulsion between the lone electron pair orbitals and halogen atoms X.

DFT scheme corrected with an empirical dispersion term (RI-TPSS-D) was used in ref. [98] for two complexes: isoflurane···leucine and isoflurane···serine···tyrosine. The DFT-RI-TPSS-D/TZVP and SAPT results show that the dispersion energy has the largest contribution to the total interaction energy.

Similar aims to investigate the nature of interactions between some anesthetic agents and protein binding sites were the motivation for ref. [100]. The authors employed three-layer ONIOM (M06-2X/6-31+G*: PM6: AMBER) method. The X-ray crystal structures were chosen as input data. This study used hydrogen and halogen bonds to perform ligand recognition and binding to the protein. According to this analysis, the polarization effects have more influence in the entire process than the steric effects. They induce a significant asymmetry in the atomic charge distributions of the non-interacting ligands. Moreover, it was concluded that water molecules are crucial for the binding of anesthetic agents to proteins, serving both as proton acceptors and proton donors, both to the hydrogen atoms and the halogen atoms of the anesthetics. Therefore, ONIOM calculations proved that water is crucial in enabling at least some anesthetic/protein interactions. This study employed a higher, more rigorous level of system description than the previous ones.

The continuation of this methodology (three-layer ONIOM: M06-2X/6-31+G*: PM6: AMBER) accompanying the Atoms in Molecules (AIM) and Electrostatic Potential (ESP) analyses was presented in paper on propofol (PFL) with three proteins (human serum albumin, GLIC and apoferritin from horse spleen) [101]. The important conclusion of this paper is that the molecular binding and anesthetic characteristic of propofol cannot be attributed solely to the hydroxyl group present in the PFL molecule. Other interactions need to be taken into account. 

New evidence is indicating that there are not only similarities but also differences between consciousness, sleep, and anesthesia. Therefore, the knowledge about neural pathways would help to lighten up and to locate the site of action of general anesthetics. In Section 4.1 we mentioned Turin et al. paper [41,42] on EPR measurement and the role of electron spin. Several experiments with high energy lasers are used to trigger the action of neurons. Consequently, the two-photon spectroscopy becomes useful and is helpful in the study of anesthetic molecules. The linear and non-linear optical properties of the halogenated ethers sevoflurane (SEVO), isoflurane (ISO) and diethyl ether were studied by Burdick et al. [42]. The authors used the entangled two-photon spectroscopy and the calculations of S_0_ → S_1_ excitation energy and highest-occupied molecular orbital-lowest unoccupied molecular orbital (HOMO-LUMO) gap by means of the TD-DFT-B3LYP with the 6-311++G(2d,p) basis set. It was found that two halogenated ethers SEVO and ISO interact with two 800 nm entangled photons, but do not interact with the same energy classical photons, while non-halogenated diethyl ether does not interact with entangled photons. These results emphasize “that individual anesthetic molecules can interact with photons” and it is probable that it is not just the interaction with macromolecules, as in the frame of the lipid bilayers and proteins concept, which is important to understand the action of anesthetics [42].

#### 4.2.3. Concept of the Role of Microtubules in Anesthetics 

In general terms, the protein target for anesthetic molecules is assumed to be membrane receptors and ion channels in neurons. Recently it was found that the interaction of volatile anesthetics with intra-neuronal microtubules is important for the understanding of the effects of anesthesia-induced postoperative cognitive dysfunction (POCD) [74,76,77,102]. Thus, a new hypothesis is now presented, saying that anesthetics could be bound to the protein subunit of microtubules, as a functional target of anesthetics.

As mentioned above, microtubules take part in maintaining the structure of eukaryotic cells. Many proteins can bind to microtubules, e.g., cytoskeletal motor proteins. The authors of [28,31] measured and calculated the collective dipole modes of oscillations in tubulin and estimated the polarizability tensors by DFT-CAM-B3LYP with the PolX basis set. The additional normal modes of aromatic amino-acids collective terahertz dipole oscillations found in the presence of the anesthetics are correlated with their anesthetic potency. The main conclusion based on cited results is as follows: anesthesia may be caused by the alteration of the dipolar oscillations of the electronic degrees of freedom in aromatic molecules in proteins of microtubules [7]. Moreover, the ability to anesthetize should be related to the polarizability and the binding affinity of anesthetics for tubulin quantum channels. This model is based on the interaction of anesthetics with tubulin and microtubules as the primary site of action.

## 5. Conclusions 

It is still difficult to elucidate the molecular mechanism of general anesthesia despite a long period of experimental medical practice. This riddle is virtually unlimited source of inspiration and discussion. In this review, we shortly described the history and the main trends in anesthesia investigation from historical roots to today’s concepts: from the lipid bilayers, through the protein targets, the inhibitory receptors NMDA, GABA_A_, to Gly and K_2p_ ion channels. We discussed recent attempts to identify specific action sites of chosen anesthetic agents: (a) the role of the non-zero nuclear spin of xenon, and (b) electron spin, as well as (c) a model employing a Radical Pair of electrons (RP) and its influence on the spin dynamics by hyperfine term due to singlet and triplet interactions, (d) anesthetics could be bound to the protein of microtubules. Finally, at the molecular level, (e) additional attention should be placed on the quantum-level description of intermolecular interaction between anesthetic molecule and receptor. Our conclusion stressed in point (e) is in agreement with the recent findings [42]. In recent years, the indication of a single molecular mechanism which could explain and describe the anesthesia has become less probable. Apparently, the state of anesthesia is likely due to many different effects and related to many different molecular targets and various types of intermolecular interactions.

## References

[1] Antkowiak B. (2001). How do general anaesthetics work?. Naturwissenschaften.

[2] Hemmings H.C., Akabas M., Goldstein P., Trudell J.R., Orser B.A., Harrison N.L. (2005). Emerging molecular mechanisms of general anesthetic action. Trends Pharmacol. Sci..

[3] Franks N.P. (2008). General anaesthesia: From molecular targets to neuronal pathways of sleep and arousal. Nat. Rev. Neurosci..

[4] Chau P.-L. (2010). New insights into the molecular mechanisms of general anaesthetics. Br. J. Pharmacol..

[5] Sear J.W. (2009). What makes a molecule an anaesthetic? Studies on the mechanisms of anaesthesia using a physicochemical approach. Br. J. Anaesth..

[6] Perouansky M., Pearce R.A., Hemmings H.C. (2010). Inhaled Anesthetics: Mechanisms of Action. Miller’s Anesth..

[7] Koch C., Massimini M., Boly M., Tononi G. (2016). Neural correlates of consciousness: Progress and problems. Nat. Rev. Neurosci..

[8] Baluška F., Yokawa K., Mancuso S., Baverstock K. (2016). Understanding of anesthesia—Why consciousness is essential for life and not based on genes. Commun. Integr. Biol..

[9] Hameroff S.R. (2006). The Entwined Mysteries of Anesthesia and Consciousness. Anesthesiology.

[10] Urban B. (2002). Current assessment of targets and theories of anaesthesia. Br. J. Anaesth..

[11] Lugli A.K., Yost C.S., Kindler C.H. (2009). Anaesthetic mechanisms: Update on the challenge of unravelling the mystery of anaesthesia. Eur. J. Anaesthesiol..

[12] Weinrich M., Worcester D.L. (2013). Xenon and Other Volatile Anesthetics Change Domain Structure in Model Lipid Raft Membranes. J. Phys. Chem. B.

[13] Son Y. (2010). Molecular mechanisms of general anesthesia. Korean J. Anesthesiol..

[14] Harris B., Moody E., Skolnick P. (1992). Isoflurane anesthesia is stereoselective. Eur. J. Pharmacol..

[15] Franks N.P., Lieb W.R. (1994). Molecular and cellular mechanisms of general anaesthesia. Nat. Cell Biol..

[16] Franks N., Lieb W.R. (1984). Do general anaesthetics act by competitive binding to specific receptors?. Nat. Cell Biol..

[17] Hutt A., Hudetz A.G. (2015). Editorial: General anesthesia: From theory to experiments. Front. Syst. Neurosci..

[18] Hales T.G., Lambert J.J. (1991). The actions of propofol on inhibitory amino acid receptors of bovine adrenomedullary chromaffin cells and rodent central neurones. Br. J. Pharmacol..

[19] Sobolevsky A.I., Rosconi M.P., Gouaux E. (2009). X-ray structure, symmetry and mechanism of an AMPA-subtype glutamate receptor. Nat. Cell Biol..

[20] Franks N., Dickinson R., De Sousa S.L.M., Hall A.C., Lieb W.R. (1998). How does xenon produce anaesthesia?. Nat. Cell Biol..

[21] Furukawa H. (2003). Mechanisms of activation, inhibition and specificity: Crystal structures of the NMDA receptor NR1 ligand-binding core. EMBO J..

[22] Dickinson R., Peterson B., Banks P., Simillis C., Martin J.C.S., Valenzuela C.A., Maze M., Franks N. (2007). Competitive Inhibition at the Glycine Site of the *N*-Methyl-d-aspartate Receptor by the Anesthetics Xenon and Isoflurane. Anesthesioloy.

[23] De Sousa S.L.M., Dickinson R., Lieb W.R., Franks N. (2000). Contrasting Synaptic Actions of the Inhalational General Anesthetics Isoflurane and Xenon. Anesthesiology.

[24] Jevtović-Todorović V., Todorovć S.M., Mennerick S., Powell S., Dikranian K., Benshoff N., Zorumski C.F., Olney J.W. (1998). Nitrous oxide (laughing gas) is an NMDA antagonist, neuroprotectant and neurotoxin. Nat. Med..

[25] Krasowski M.D., Harrison N.L. (2000). The actions of ether, alcohol and alkane general anaesthetics on GABAA and glycine receptors and the effects of TM2 and TM3 mutations. Br. J. Pharmacol..

[26] Patel A.J., Honore E., Lesage F., Fink M., Romey G., Lazdunski M. (1999). Inhalational anesthetics activate two-pore-domain background K+ channels. Nat. Neurosci..

[27] Pickholz M., Saiz L., Klein M.L. (2005). Concentration Effects of Volatile Anesthetics on the Properties of Model Membranes: A Coarse-Grain Approach. Biophys. J..

[28] Travis J.A.C., Stuart R.H., Ahmed T.A., Mariusz K., Jack A.T. (2015). Anesthetics Act in Quantum Channels in Brain Microtubules to Prevent Consciousness. Curr. Top. Med. Chem..

[29] Arvayo-Zatarain J.A., Favela-Rosales F., Contreras-Aburto C., Urrutia-Bañuelos E., Maldonado A. (2018). Molecular dynamics simulation study of the effect of halothane on mixed DPPC/DPPE phospholipid membranes. J. Mol. Model..

[30] Arcario M., Mayne C.G., Tajkhorshid E. (2014). Atomistic Models of General Anesthetics for Use in in Silico Biological Studies. J. Phys. Chem. B.

[31] Craddock T.J.A., Kurian P., Preto J., Sahu K., Hameroff S.R., Klobukowski M., Tuszynski J. (2017). Anesthetic Alterations of Collective Terahertz Oscillations in Tubulin Correlate with Clinical Potency: Implications for Anesthetic Action and Post-Operative Cognitive Dysfunction. Sci. Rep..

[32] Pavel M.A., Petersen E.N., Wang H., Lerner R.A., Hansen S.B. (2020). Studies on the mechanism of general anesthesia. Proc. Natl. Acad. Sci. USA.

[33] Kohn W., Sham L.J. (1965). Self-Consistent Equations Including Exchange and Correlation Effects. Phys. Rev..

[34] Jeziorski B., Moszynski R., Szalewicz K. (1994). Perturbation Theory Approach to Intermolecular Potential Energy Surfaces of van der Waals Complexes. Chem. Rev..

[35] Aidas K., Angeli C., Bak K.L., Bakken V., Bast R., Boman L., Christiansen O., Cimiraglia R., Coriani S., Dahle P. (2014). The Dalton quantum chemistry program system. Wiley Interdiscip. Rev. Comput. Mol. Sci..

[36] Olsen J.M., Reine S., Vahtras O., Kjellgren E., Reinholdt P., Dundas K.O.H.M., Li X., Cukras J., Ringholm M., Hedegård E.D. (2020). Dalton Project: A Python platform for molecular- and electronic-structure simulations of complex systems. J. Chem. Phys..

[37] Bondanza M., Nottoli M., Cupellini L., Lipparini F., Mennucci B. (2020). Polarizable embedding QM/MM: The future gold standard for complex (bio)systems?. Phys. Chem. Chem. Phys..

[38] Řezáč J., Riley K.E., Hobza P. (2011). S66: A Well-balanced Database of Benchmark Interaction Energies Relevant to Biomolecular Structures. J. Chem. Theory Comput..

[39] Verma P., Truhlar D. (2020). Status and Challenges of Density Functional Theory. Trends Chem..

[40] Li N., Lu D., Yang L., Tao H., Xu Y., Wang C., Fu L., Liu H., Chummum Y., Zhang S. (2018). Nuclear Spin Attenuates the Anesthetic Potency of Xenon Isotopes in Mice. Anesthesiology.

[41] Turin L., Skoulakis E.M.C., Horsfield A.P. (2014). Electron spin changes during general anesthesia in Drosophila. Proc. Natl. Acad. Sci. USA.

[42] Burdick R.K., Villabona-Monsalve J.P., Mashour G.A., Goodson T. (2019). Modern Anesthetic Ethers Demonstrate Quantum Interactions with Entangled Photons. Sci. Rep..

[43] Cohen A., Mori-Sánchez P., Yang W. (2012). Challenges for Density Functional Theory. Chem. Rev..

[44] Gundelach L., Fox T., Tautermann C.S., Skylaris C.-K. (2021). Protein–ligand free energies of binding from full-protein DFT calculations: Convergence and choice of exchange–correlation functional. Phys. Chem. Chem. Phys..

[45] Kodrycka M., Holzer C., Klopper W., Patkowski K. (2019). Explicitly Correlated Dispersion and Exchange Dispersion Energies in Symmetry-Adapted Perturbation Theory. J. Chem. Theory Comput..

[46] Garcia J., Podeszwa R., Szalewicz K. (2020). SAPT codes for calculations of intermolecular interaction energies. J. Chem. Phys..

[47] Smith D.G.A., Burns L.A., Simmonett A.C., Parrish R.M., Schieber M.C., Galvelis R., Kraus P., Kruse H., Di Remigio R., Alenaizan A. (2020). Psi4 1.4: Open-source software for high-throughput quantum chemistry. J. Chem. Phys..

[48] Werner H.-J., Knowles P.J., Manby F.R., Black J.A., Doll K., Heßelmann A., Kats D., Köhn A., Korona T., Kreplin D.A. (2020). The Molpro quantum chemistry package. J. Chem. Phys..

[49] Parrish R.M., Parker T.M., Sherrill C.D. (2014). Chemical Assignment of Symmetry-Adapted Perturbation Theory Interaction Energy Components: The Functional-Group SAPT Partition. J. Chem. Theory Comput..

[50] Parrish R.M., Sherrill D. (2014). Spatial assignment of symmetry adapted perturbation theory interaction energy components: The atomic SAPT partition. J. Chem. Phys..

[51] Misquitta A., Podeszwa R., Jeziorski B., Szalewicz K. (2005). Intermolecular potentials based on symmetry-adapted perturbation theory with dispersion energies from time-dependent density-functional calculations. J. Chem. Phys..

[52] Jansen G. (2014). Symmetry-adapted perturbation theory based on density functional theory for noncovalent interactions. Wiley Interdiscip. Rev. Comput. Mol. Sci..

[53] Heßelmann A., Jansen G., Schütz M. (2005). Density-functional theory-symmetry-adapted intermolecular perturbation theory with density fitting: A new efficient method to study intermolecular interaction energies. J. Chem. Phys..

[54] Yamakura T., Harris R.A. (2000). Effects of Gaseous Anesthetics Nitrous Oxide and Xenon on Ligand-gated Ion Channels. Anesthesiology.

[55] Nunzi F., Pannacci G., Tarantelli F., Belpassi L., Cappelletti D., Falcinelli S., Pirani F. (2020). Leading Interaction Components in the Structure and Reactivity of Noble Gases Compounds. Molecules.

[56] Yamamoto E., Akimoto T., Shimizu H., Hirano Y., Yasui M., Yasuoka K. (2012). Diffusive Nature of Xenon Anesthetic Changes Properties of a Lipid Bilayer: Molecular Dynamics Simulations. J. Phys. Chem. B.

[57] Liu L.T., Xu Y., Tang P. (2010). Mechanistic Insights into Xenon Inhibition of NMDA Receptors from MD Simulations. J. Phys. Chem. B.

[58] Koziakova M., Harris K., Edge C.J., Franks N., White I.L., Dickinson R. (2019). Noble gas neuroprotection: Xenon and argon protect against hypoxic–ischaemic injury in rat hippocampus in vitro via distinct mechanisms. Br. J. Anaesth..

[59] Chen J., Chen L., Wang Y., Wang X., Zeng S. (2015). Exploring the Effects on Lipid Bilayer Induced by Noble Gases via Molecular Dynamics Simulations. Sci. Rep..

[60] Petrenko A.B., Yamakura T., Sakimura K., Baba H. (2014). Defining the role of NMDA receptors in anesthesia: Are we there yet?. Eur. J. Pharmacol..

[61] Andrijchenko N.N., Ermilov A.Y., Khriachtchev L., Räsänen M., Nemukhin A.V. (2014). Toward Molecular Mechanism of Xenon Anesthesia: A Link to Studies of Xenon Complexes with Small Aromatic Molecules. J. Phys. Chem. A.

[62] Dzięcioł B., Gańko S., Cukras J.

[63] Smith J., Haghighi H.Z., Salahub D., Simon C. (2021). Radical pairs may play a role in xenon-induced general anesthesia. Sci. Rep..

[64] Babcock N.S., Kattnig D.R. (2020). Electron—Electron Dipolar Interaction Poses a Challenge to the Radical Pair Mechanism of Magnetoreception. J. Phys. Chem. Lett..

[65] Bradlaugh A., Munro A.L., Jones A.R., Baines R.A. (2021). Exploiting the Fruitfly, *Drosophila melanogaster*, to Identify the Molecular Basis of Cryptochrome-Dependent Magnetosensitivity. Quantum Rep..

[66] Turin L., Skoulakis E.M. (2018). Electron Spin Resonance (EPR) in Drosophila and General Anesthesia. Methods in Enzymology.

[67] Hiscock H.G., Mouritsen H., Manolopoulos D.E., Hore P. (2017). Disruption of Magnetic Compass Orientation in Migratory Birds by Radiofrequency Electromagnetic Fields. Biophys. J..

[68] Stenqvist O., Husum B., Dale O. (2001). Nitrous oxide: An ageing gentleman. Acta Anaesthesiol. Scand..

[69] Brown S., Sneyd J. (2016). Nitrous oxide in modern anaesthetic practice. BJA Educ..

[70] Emmanouil D.E., Quock R.M. (2007). Advances in Understanding the Actions of Nitrous Oxide. Anesthesia Prog..

[71] Becker D.E., Rosenberg M. (2008). Nitrous Oxide and the Inhalation Anesthetics. Anesthesia Prog..

[72] de Vasconcellos K. (2010). Nitrous oxide in 2010: Who will have the last laugh? (Part 1). S. Afr. J. Anaesth. Analg..

[73] Eagleman S.L., Drover C.M., Drover D.R., Ouellette N.T., Maciver M.B. (2018). Remifentanil and Nitrous Oxide Anesthesia Produces a Unique Pattern of EEG Activity During Loss and Recovery of Response. Front. Hum. Neurosci..

[74] Nagele P., Metz L.B., Crowder C.M. (2004). Nitrous oxide (N_2_O) requires the N-methyl-D-aspartate receptor for its action in Caenorhabditis elegans. Proc. Natl. Acad. Sci. USA.

[75] Buhre W., Disma N., Hendrickx J., DeHert S., Hollmann M.W., Huhn R., Jakobsson J., Nagele P., Peyton P., Vutskits L. (2019). European Society of Anaesthesiology Task Force on Nitrous Oxide: A narrative review of its role in clinical practice. Br. J. Anaesth..

[76] Iqbal F., Thompson A.J., Riaz S., Pehar M., Rice T., Syed N.I. (2019). Anesthetics: From modes of action to unconsciousness and neurotoxicity. J. Neurophysiol..

[77] Kalmoe M.C., Janski A.M., Zorumski C.F., Nagele P., Palanca B.J., Conway C.R. (2020). Ketamine and nitrous oxide: The evolution of NMDA receptor antagonists as antidepressant agents. J. Neurol. Sci..

[78] Emmanouil D. (2020). Mechanism of Action of Nitrous Oxide. Nitrous Oxide in Pediatric Dentistry.

[79] Lew V., McKay E., Maze M. (2018). Past, present, and future of nitrous oxide. Br. Med Bull..

[80] Philip N.S., Carpenter L.L., Tyrka A.R., Price L.H. (2010). Nicotinic acetylcholine receptors and depression: A review of the preclinical and clinical literature. Psychopharmacology.

[81] Sadlej J., Siciński M. (1990). Investigations of the anaesthetic activity of nitrous oxide by quantum-chemical calculations. J. Mol. Struct. THEOCHEM.

[82] Berner G.M., East A.L.L., Afshari M., Dehghany M., Moazzen-Ahmadi N., McKellar A.R.W. (2009). Nitrous oxide dimer: An ab initio coupled-cluster study of isomers, interconversions, and infrared fundamental bands, and experimental observation of a new fundamental for the polar isomer. J. Chem. Phys..

[83] Valdes H., Sordo J.A. (2004). The N_2_O·N_2_O, N_2_O·SO_2_, and (N_2_O)_2_·SO_2_ van der Waals Complexes: An ab Initio Theoretical Analysis. J. Phys. Chem. A.

[84] Arcario M.J., Mayne C.G., Tajkhorshid E. (2017). A membrane-embedded pathway delivers general anesthetics to two interacting binding sites in the Gloeobacter violaceus ion channel. J. Biol. Chem..

[85] Vemparala S., Domene C., Klein M.L. (2010). Computational Studies on the Interactions of Inhalational Anesthetics with Proteins. Accounts Chem. Res..

[86] Bjelkmar P., Larsson P., Cuendet M.A., Hess B., Lindahl E. (2010). Implementation of the CHARMM Force Field in GROMACS: Analysis of Protein Stability Effects from Correction Maps, Virtual Interaction Sites, and Water Models. J. Chem. Theory Comput..

[87] (2021). Anesthetic May Affect Tau Spread in the Brain to Promote Alzheimer’s Disease Pathology, Neuroscience News. https://neurosciencenews.com/anesthetic-tau-alzheimers-18434/.

[88] Stock L., Hosoume J., Cirqueira L., Treptow W. (2018). Binding of the general anesthetic sevoflurane to ion channels. PLoS Comput. Biol..

[89] Lipiński P.F.J., Jarończyk M., Dobrowolski J.C., Sadlej J. (2019). Molecular dynamics of fentanyl bound to μ-opioid receptor. J. Mol. Model..

[90] Faulkner C., de Leeuw N.H. (2020). In silico studies of the interactions between propofol and fentanyl using Gaussian accelerated molecular dynamics. J. Biomol. Struct. Dyn..

[91] Faulkner C., Plant D.F., De Leeuw N.H. (2019). Modulation of the Gloeobacter violaceus Ion Channel by Fentanyl: A Molecular Dynamics Study. Biochemistry.

[92] Faulkner C., Santos-Carballal D., Plant D.F., De Leeuw N.H. (2020). Atomistic Molecular Dynamics Simulations of Propofol and Fentanyl in Phosphatidylcholine Lipid Bilayers. ACS Omega.

[93] Wague A., Joseph T., Woll K.A., Bu W., Vaidya K.A., Bhanu N.V., Garcia B.A., Nimigean C.M., Eckenhoff R.G., Riegelhaupt P.M. (2020). Mechanistic insights into volatile anesthetic modulation of K2P channels. eLife.

[94] Dapprich S., Komáromi I., Byun K., Morokuma K., Frisch M.J. (1999). A new ONIOM implementation in Gaussian98. Part I. The calculation of energies, gradients, vibrational frequencies and electric field derivatives. J. Mol. Struct. THEOCHEM.

[95] Vreven T., Byun K.S., Komáromi I., Dapprich S., Montgomery J., Morokuma K., Frisch M.J. (2006). Combining Quantum Mechanics Methods with Molecular Mechanics Methods in ONIOM. J. Chem. Theory Comput..

[96] Zierkiewicz W., Wieczorek R., Hobza P., Michalska D. (2011). Halogen bonded complexes between volatile anaesthetics (chloroform, halothane, enflurane, isoflurane) and formaldehyde: A theoretical study. Phys. Chem. Chem. Phys..

[97] Zierkiewicz W., Michalska D., Zeegers-Huyskens T. (2013). Theoretical studies of the interaction between enflurane and water. J. Mol. Model..

[98] Zierkiewicz W. (2012). Nature of multiple weak interactions between volatile anaesthetic isoflurane and apoferritin: A theoretical study. Chem. Phys..

[99] Kolář M.H., Hobza P. (2016). Computer Modeling of Halogen Bonds and Other σ-Hole Interactions. Chem. Rev..

[100] Qiu L., Lin J., Bertaccini E.J. (2015). Insights into the Nature of Anesthetic–Protein Interactions: An ONIOM Study. J. Phys. Chem. B.

[101] Qiu L., Lin J., Liu Q., Wang S., Lv G., Li K., Shi H., Huang Z., Bertaccini E.J. (2017). The Role of the Hydroxyl Group in Propofol–Protein Target Recognition: Insights from ONIOM Studies. J. Phys. Chem. B.

[102] Hameroff S. (1998). Anesthesia, consciousness and hydrophobic pockets—A unitary quantum hypothesis of anesthetic action. Toxicol. Lett..

